# Association of triglyceride-glucose index with cardiovascular disease among a general population: a prospective cohort study

**DOI:** 10.1186/s13098-023-01181-z

**Published:** 2023-10-16

**Authors:** Yiming Wan, Ziliang Zhang, Yong Ling, Hui Cui, Zihan Tao, Jianfeng Pei, Aikedan Maimaiti, Haifan Bai, Yiling Wu, Jing Li, Genming Zhao, Maryam Zaid

**Affiliations:** 1 Department of Epidemiology, School of Public Health, Fudan University, Shanghai, China; 2 Shanghai Depeac Biotechnology Co., Ltd, Shanghai, China; 3 Department of Plastic Surgery, Changhai Hospital, Naval Medical University, Shanghai, China; 4 Songjiang Center for Disease Control and Prevention, Shanghai, China; 5 Songjiang District Zhongshan Street Community Healthcare Center, Shanghai, China

**Keywords:** Triglyceride-glucose index, Cardiovascular disease, Cohort studies

## Abstract

**Background:**

The impact of triglyceride-glucose (TyG) index, a surrogate marker for insulin resistance, on the risk of cardiovascular disease (CVD) in general populations remains controversial. We aimed to comprehensively study the relationship between TyG index with the risk of incident CVD events in the general population in Shanghai.

**Methods:**

A total of 42,651 participants without previous history of CVD events from Shanghai Suburban Adult Cohort and Biobank (SSACB) were included. SSACB was a community-based natural population cohort study using multistage cluster sampling method. TyG index was calculated as Ln [fasting serum triglyceride (mg/dL) * fasting blood glucose (mg/dL)/2]. Kaplan-Meier curves, log-rank test and cox proportional hazards model were used to calculate the association between TyG index and incident CVD, including stroke and coronary heart disease (CHD). Restricted cubic spline analyses were used to determine whether there was a non-linear relationship between TyG index and CVD events.

**Results:**

During a median follow-up of 4.7 years, 1,422 (3.3%) individuals developed CVD, including 674 (1.6%) cases of stroke and 732 (1.7%) cases of CHD. A one unit increment higher TyG index was associated with [HR(95%CI)] 1.16(1.04–1.29) in CVD and with 1.39(1.19–1.61) in stroke. Only linear relationships between TyG and CVD/stroke were observed, while no relationship was observed with CHD after adjustments for confounders. In subgroup analyses, younger (< 50y) and diabetic participants had higher risk of CVD than their counterpart groups, while hypertensive and dyslipidemic participants depicted lower risks than their counterparts.

**Conclusion:**

Elevated TyG index was associated with a higher risk of incident CVD and stroke. TyG index may help in the early stage of identifying people at high risk of CVD.

**Supplementary Information:**

The online version contains supplementary material available at 10.1186/s13098-023-01181-z.

## Background

Cardiovascular disease (CVD), including stroke and coronary heart disease (CHD), is one of the leading causes of mortality worldwide, contributing to an estimated 17.9 million deaths every year [1]. CVD has resulted in serious public health challenges and has caused huge economic burdens [2]. During the past few decades, due to rapid transitions in demography, epidemiology and lifestyles, CVD has been the primary cause of death in China [3]. Although several risk factors for CVD have been determined, recent studies have demonstrated that some individuals without known risk factors may also develop CVD [4].

Insulin resistance (IR), a pathophysiological condition of decreased sensitivity and responsiveness to insulin, has been recognized as a characteristic of metabolic syndrome (MS) and atherosclerosis [5, 6]. There are two golden standards of IR detection, the euglycemic insulin clamp and intravenous glucose tolerance testing, but due to their invasiveness and high cost, they are not used in clinical areas [7]. Although the homeostasis model assessment estimated insulin resistance (HOMA-IR) index has been widely used, its use is limited when individuals are under insulin treatment or are without functioning beta cells [7]. The triglyceride-glucose (TyG) index, a logarithmized product of triglyceride (TG) and fasting plasma glucose (FPG), is a convenient and low-cost way to detect IR and has been demonstrated to be superior to HOMA-IR [8]. TyG index is calculated by ln [fasting serum TG (mg/dL) × FPG (mg/dL)/2] [8]. Higher values of TyG index demonstrate a greater degree of IR and reflect disturbances in both glucose and lipid metabolism [9]. Several cross-sectional studies have indicated that TyG index is associated with the incidence of CVD [10–13]. The results from Vascular Metabolic CUN (VMCUN) cohort first found that there was a positive association between TyG index and CVD events [14]. Moreover, a meta-analysis combining eight different cohorts also showed that a higher TyG index might be related to a higher risk of developing CVD, independent of age, sex, and diabetic status [15]. Studies in China involved participants from Kailuan community in Tangshan, where over 75% were male [16, 17]. To date, there were no more prospective cohort studies based on a general population in China. Therefore, large community-based cohort studies are needed to verify the association between TyG index and future CVD events, especially in a general population. For TyG index, as a compound index, we hypothesized that it can help to predict CVD in a Chinese general population.

Using data from the Shanghai Suburban Adult Cohort and Biobank (SSACB), we aimed to comprehensively study the relationship between TyG index with the risk of incident CVD events.

## Methods

### Study population

The SSACB study is a large-scale community-based natural cohort study in Eastern China. From June 2016 to December 2017, baseline information was collected from four communities in Songjiang district (Zhongshan, Xinqiao, Sheshan, and Maogang) and three in Jiading district (Anting, Huating, and Huangdu). All the participants from Songjiang and Jiading districts were recruited through using multistage cluster sampling method and based on their willingness, health service facilities, geographic region and electronic medical record system. [18] From September 2018 to January 2020, information was collected from Minhang and Xuhui districts. Details of the SSACB cohort have been published previously [18]. The last day of follow-up was Jan 31st, 2022 for participants in Songjiang district and Feb 26th, 2022 for those in Jiading district. This study was constructed under the approval of the ethical review board of the School of Public Health of Fudan University (IRB#2016-04-0586). Informed consent was obtained from all participants of the SSACB cohort.

**Fig. 1:**
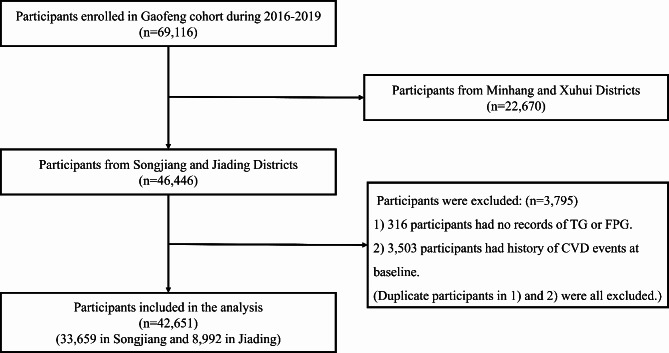
Flow diagram of the inclusion and exclusion of participants from SSACB

In our analysis, we included participants who have lived in Shanghai for at least 5 years aged 20–74 years old at baseline in 2016–2017. Of 69,116 participants recruited at baseline, we excluded participants in the Minhang district and Xuhui district (n = 22,670), those who did not have data on TG or FPG (n = 316), and those who had a history of CVD events (n = 3,503). Duplicate participants (n = 24) were all excluded. After exclusion, 42,651 eligible participants were included in our analysis, including 33,659 in the Songjiang district and 8,992 in the Jiading district. (Fig. 1)

### Data collection

We used baseline information from face-to-face interviews and from clinical examinations conducted by well-trained staff using standardized and validated instruments. From interviews, information on demographic characteristics (e.g., age, sex, educational level, marriage status, socioeconomic status and retirement status), lifestyle factors (e.g., smoking status, drinking status and physical activity) and history of diseases (e.g., CVD, hypertension, and diabetes) were collected. The short form of International Physical Activity Questionnaire (IPAQ) was used to assess physical activity level. Anthropometric parameters including height, weight, and blood pressure were measured at the local community health center. Blood and urine samples were collected and tested, providing information on total cholesterol (TC), high-density lipoprotein cholesterol (HDL-C), low-density lipoprotein cholesterol (LDL-C), TG, FPG, and glycated hemoglobin (HbA1c). Health records of each participant, including the use of antihypertensive medication, antidiabetic medication, and diagnosis of diseases and deaths, were obtained from the Electronic Medical Record System (EMR), the Cardiovascular and Cerebrovascular Disease Registration and Reporting System (CCDRR), and the Cause-of-Death Surveillance System (CDSS) with unique numbers.

### Data definitions

Education level was categorized into three groups: primary school and below, middle school, and high school and above. Smoking status was defined as current smokers (those who smoked at least one cigarette per day for at least 6 months) or not. Drinking status was defined as current drinkers (those who drank more than three times per week for at least 6 months) or not. Physical activity was categorized into low, moderate, and high levels according to the Guidelines for Data processing and Analysis of the IPAQ, using the metabolic equivalent (MET). MET was calculated using different MET coefficients of various activities [19]. Body Mass Index (BMI) was calculated using the formula: weight (kg) / height^2^ (m^2^), and categorized into four groups: underweight (< 18.5 kg/m^2^), normal (18.5–23.9 kg/m^2^), overweight (24-27.9 kg/m^2^) and obese (≥ 28 kg/m^2^) according to the standard of Chinese body mass index [20]. Hypertension was defined as self-report hypertension, systolic blood pressure (SBP) ≥ 140 mmHg or diastolic blood pressure (DBP) ≥ 90 mmHg, or use of antihypertensive medication. Diabetes was defined as self-report diabetes or FPG ≥ 7.0 mmol/L. Dyslipidemia was defined as self-report dyslipidemia or a combination of TC level ≥ 240 mg/dL (6.20 mmol/L), LDL-C level ≥ 160 mg/dL (4.13 mmol/L), TG level ≥ 200 mg/dL (2.25 mmol/L) or HDL-C level < 40 mg/dL (1.03 mmol/L) [21].

TyG index was calculated by the following formula: ln [fasting serum TG (mg/dL) × FPG (mg/dL)/2] [8]. To convert TG from mmol/L to mg/dL, divide by 0.01129. To convert FPG from mmol/L to mg/dL, multiply by 18.

### Definition of outcomes

The outcome of this study was incident CVD events, defined as fatal or non-fatal stroke or CHD according to the International Statistical Classification of Diseases and Related Health Problems, Tenth Revision (ICD-10). Stroke was defined as a confirmed diagnosis, such as subarachnoid or intracerebral hemorrhage, other nontraumatic intracranial hemorrhage, cerebral infarction, or stroke, not specified as hemorrhage or infarction (ICD-10 I60 to I64). CHD was defined as a confirmed diagnosis, which includes angina pectoris, acute myocardial infarction, subsequent myocardial infarction, certain current complications following acute myocardial infarction, other acute ischaemic heart diseases, and chronic ischaemic heart disease (ICD-10 I20 to I25). The definition of atherosclerotic cardiovascular diseases (ASCVD) was ischaemic heart diseases (ICD-10 I63-I64) and CHD (ICD-10 I20-I25). Information on CVD events and deaths was collected through CCDRR and CDSS.

### Statistical analysis

Baseline characteristics of participants were expressed according to quartiles of TyG index. Continuous variables with bell-shaped distributions were presented as means ± standard deviation (SD) and those without bell-shaped distributions were presented as median (interquartile range). Categorical variables were presented as a number of cases (percentage). Trends across quartiles of TyG index were calculated using generalized linear regression analysis for continuous variables and the Cochran-Armitage trend chi-square test for categorical variables. The relationships between quartiles of TyG index and incident CVD events, stroke, and CHD were performed by Kaplan-Meier curves and log-rank test. Cox proportional hazards model was used to calculate the association between TyG index and incident outcomes, estimating the hazard ratios (HRs) and 95% confidence intervals (CIs). TyG index was modelled either as a continuous variable or as a categorical variable (as quartiles of TyG index). Four models were established: Model 1 was unadjusted; Model 2 was adjusted for age at baseline (years) and sex; Model 3 was further adjusted for BMI, education level, physical activity, current smoking, and current drinking; Model 4 was further adjusted for HDL-C (mmol/L), uric acid (µmol/L), use of antihypertensive medication and use of antidiabetic medication. In order to determine whether there was a non-linear relationship between TyG index and outcomes, restricted cubic splines (RCS) with Cox proportional hazards modelling were applied. Four knots were used in RCS curves: 5th, 35th, 65th, and 95th of TyG index, in which the 35th knot was used as the reference. Subgroup analyses aimed to determine whether the association between TyG index with different CVD outcomes differed by sex (male or female), age (< 50 years or ≥ 50 years), BMI groups (< 18.5 kg/m^2^, 18.5–23.9 kg/m^2^, 24-27.9 kg/m^2^ or ≥ 28 kg/m^2^), hypertension (yes or no), diabetes (yes or no) and dyslipidemia (yes or no). Interaction tests were conducted by adding a product of TyG index and subgroup into Model 4. Additionally, we assessed the relationship between TyG index and incidence of ASCVD, separating stroke into ischaemic stroke and haemorrhagic stroke. In sensitivity analysis, we excluded CVD cases within the first year during the follow-up period. All statistical analyses were conducted by SAS statistical software version 9.4 (SAS Institute, Cary, NC, USA). *P* values less than 0.05 (two-tailed) were considered as statistically significant.

## Results

### Baseline characteristics

The baseline characteristics of participants both overall and according to quartiles of TyG index were presented in Table 1. Of the 42,651 participants, 25,447 (59.7%) were women and the mean age at baseline was 55.7 ± 11.1 years. The median TyG index was 8.6 (8.2, 9.0). The prevalence of hypertension, diabetes, and dyslipidemia were 50.0%, 10.2% and 34.6%, respectively. Participants in the highest quartile of TyG index were less likely to be women, and more likely to be older, current smokers, and drinkers. Moreover, a higher TyG index was associated with higher BMI, blood pressure, uric acid, FPG, TG, TC, HbA1c, and lower HDL-C. A higher rate of antihypertensive medication use and antidiabetic medication use was observed with higher TyG levels. Baseline characteristics of the study population according to CVD, stroke, and CHD cases, in comparison to healthy participants are shown in Table S1.

**Table 1:** Baseline characteristics of the study population both overall and according to quartiles of TyG index

Characteristics^a^	TyG index^b^					*P* for trend^c^
	Total	Q1 (6.53–8.25)	Q2 (8.25–8.61)	Q3 (8.61–9.02)	Q4 (9.02–12.38)	
N	42,651	10,662	10,663	10,663	10,663	
Female	25,447 (59.7)	6,632 (62.2)	6,527 (61.2)	6,423 (60.2)	5,865 (55.0)	< 0.001
Age, years	55.7 ± 11.1	53.0 ± 13.0	55.8 ± 11.1	56.8 ± 10.0	57.2 ± 9.5	< 0.001
Education level						
Primary school and below	5,180 (12.2)	1,122 (10.5)	1,332 (12.5)	1,329 (12.5)	1,397 (13.1)	< 0.001
Middle school	12,422 (29.1)	2,977 (27.9)	3,167 (29.7)	3,234 (30.3)	3,044 (28.6)	
High school or above	25,049 (58.7)	6,563 (61.6)	6,164 (57.8)	6,100 (57.2)	6,222 (58.4)	
Current smoking	10,395 (24.4)	2,282 (21.4)	2,476 (23.2)	2,566 (24.1)	3,071 (28.8)	< 0.001
Current drinking	5,758 (13.5)	1,316 (12.3)	1,306 (12.3)	1,397 (13.1)	1,739 (16.3)	< 0.001
Physical activity level						
Low	4,173 (9.8)	1,007 (9.4)	996 (9.3)	1,029 (9.7)	1,141 (10.7)	< 0.001
Moderate	13,803 (32.4)	3,707 (34.8)	3,369 (31.6)	3,304 (31.0)	3,423 (32.1)	
High	24,675 (57.9)	5,948 (55.8)	6,298 (59.1)	6,330 (59.4)	6,099 (57.2)	
Body mass index, kg/m^2^	24.2 ± 3.3	22.8 ± 3.1	23.8 ± 3.2	24.7 ± 3.2	25.6 ± 3.1	< 0.001
Systolic blood pressure, mmHg	132.8 ± 19.3	127.0 ± 18.3	131.4 ± 18.8	134.5 ± 18.9	138.5 ± 19.4	< 0.001
Diastolic blood pressure, mmHg	79.6 ± 10.6	76.8 ± 10.2	79.0 ± 10.4	80.5 ± 10.3	82.6 ± 10.6	< 0.001
Uric acid, mg/dL	303.8 ± 80.7	276.8 ± 71.0	293.1 ± 74.0	310.8 ± 78.5	334.3 ± 86.8	< 0.001
Fasting plasma glucose, mmol/L	4.9 (4.4, 5.5)	4.5 (4.2, 4.8)	4.8 (4.3, 5.2)	5.0 (4.5, 5.5)	5.5 (4.9, 6.5)	< 0.001
Triglycerides, mmol/L	1.4 (1.0, 2.0)	0.8 (0.7, 0.9)	1.2 (1.1, 1.3)	1.7 (1.5, 1.9)	2.6 (2.2, 3.4)	< 0.001
Total cholesterol, mmol/L	4.9 ± 0.9	4.6 ± 0.8	4.9 ± 0.8	5.0 ± 0.9	5.3 ± 1.0	< 0.001
High-density lipoprotein cholesterol, mmol/L	1.4 ± 0.4	1.6 ± 0.4	1.5 ± 0.3	1.4 ± 0.3	1.2 ± 0.3	< 0.001
Low-density lipoprotein cholesterol, mmol/L	2.8 ± 0.8	2.6 ± 0.7	2.8 ± 0.8	2.9 ± 0.8	2.7 ± 1.0	< 0.001
HbA1c, %	5.7 (5.4, 6.0)	5.5 (5.2, 5.7)	5.6 (5.3, 5.9)	5.7 (5.4, 6.0)	5.9 (5.6, 6.5)	< 0.001
Hypertension	21,342 (50.0)	3,621 (34.0)	4,855 (45.5)	5,806 (54.5)	7,060 (66.2)	< 0.001
Antihypertensive medication	11,786 (27.6)	1,861 (17.5)	2,582 (24.2)	3,246 (30.4)	4,097 (38.4)	< 0.001
Diabetes	4,357 (10.2)	287 (2.7)	472 (4.4)	932 (8.7)	2,666 (25.0)	< 0.001
Antidiabetic medication	4,538 (10.6)	342 (3.2)	582 (5.5)	1,079 (10.1)	2,535 (23.8)	< 0.001
Dyslipidemia	14,770 (34.6)	938 (8.8)	1,786 (16.8)	3,342 (31.3)	8,704 (81.6)	< 0.001

N, number; Q, quartiles; TyG, triglyceride-glucose; HbA1c, glycated hemoglobin A1c

^a^ Continuous variables were described as mean ± standard deviation (SD); Categorical variables were described as frequency (percentage)

^b^ Exact values of quartiles of TyG index are as follows: (Q1) ≤ 8.246, (Q2) ≤ 8.609 and > 8.246, (Q3) ≤ 9.020 and > 8.609, and (Q4) > 9.020

^c^ For continuous variables, *P* for trend across quartiles of TyG index were calculated by the generalized linear regression analysis; for categorical variables, *P* for trend were calculated by the Cochran-Armitage trend chi-square test

### Association of TyG index with incident CVD events

During a median follow-up of 4.7 years (204,748.6 person-years), 1,422 (3.3%) participants developed CVD, including 674 (1.6%) cases of stroke and 732 (1.7%) cases of CHD.

**Fig. 2:**
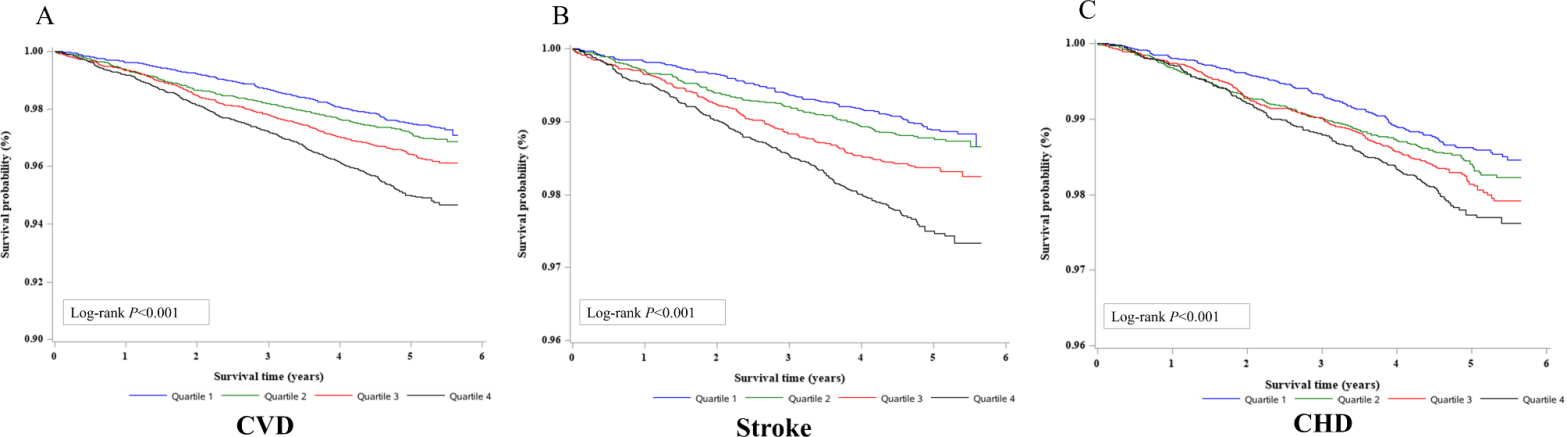
Kaplan-Meier curves of incident cardiovascular disease according to quartiles of TyG index. TyG, triglyceride-glucose. Exact values of quartiles of TyG index are as follows: (Q1) ≤ 8.246, (Q2) ≤ 8.609 and > 8.246, (Q3) ≤ 9.020 and > 8.609, and (Q4) > 9.020

The Kaplan-Meier curves of CVD according to the quartiles of TyG index demonstrated that a higher quartile of TyG index was associated with a higher risk of developing CVD events (all Log-rank *P* < 0.001, Fig. 2). Table 2 showed the association between TyG index with risk of CVD events. The incidence rates per 1000 person-years of CVD, stroke and CHD were generally higher in higher quartiles of TyG index. In the unadjusted Cox proportional model (Model 1), the highest and third quartiles of TyG index were correlated with an increased risk of developing CVD, stroke, and CHD compared to the lowest quartile (*P* for trend < 0.001). After adjusting for age and sex (Model 2), the trends persisted only in CVD and stroke. With CHD, only the highest quartile of TyG index was found to be associated with higher risk [HR (95%CI): 1.41 (1.14–1.73)] compared to the lowest quartile (*P* for trend < 0.001). In a further adjusted model (Model 3), similar trends were observed in CVD and stroke, but not in CHD. After adjusting for all covariates (Model 4), higher quartiles of TyG index maintained an increased risk with stroke, although HRs were slightly attenuated. No relationship was found with quartiles of TyG index and CHD in the final model. Linear relationships between TyG index and CVD and stroke were maintained, which indicated that each unit increment in TyG index was associated with 16% and 39% increase in the risk of CVD and stroke, respectively. However, no linear relationship was observed between TyG index and CHD after full adjustment for covariates (Model 4). In sensitivity analyses, after excluding CVD events that occurred within the first year of follow-up, similar results were observed in the associations between TyG index with risk of CVD, stroke, and CHD (Table S2).

**Table 2:** Association between TyG index and incidence of cardiovascular diseases

Event	TyG	No. of cases/population	Incidence rate per 1000 person-years	Hazard ratio (95% CI)			
				Model 1	Model 2	Model 3	Model 4
CVD	Per 1 unit	1,422/42,651		1.54 (1.43–1.67)***	1.51 (1.39–1.63)***	1.43 (1.31–1.56)***	1.16 (1.04–1.29)**
	Q1	262/10,662	4.95	1.00 (Reference)	1.00 (Reference)	1.00 (Reference)	1.00 (Reference)
	Q2	296/10,663	5.72	1.16 (0.98–1.37)	1.07 (0.90–1.26)	1.01 (0.85–1.20)	0.95 (0.80–1.13)
	Q3	368/10,663	7.21	1.46 (1.24–1.71)***	1.32 (1.13–1.55)***	1.22 (1.04–1.44)*	1.06 (0.89–1.25)
	Q4	496/10,663	9.88	2.00 (1.72–2.32)***	1.80 (1.55–2.10)***	1.62 (1.39–1.90)***	1.18 (0.98–1.41)
Stroke	Per 1 unit	674/42,651		1.73 (1.55–1.93)***	1.71 (1.52–1.91)***	1.65 (1.46–1.86)***	1.39 (1.19–1.61)***
	Q1	115/10,662	2.16	1.00 (Reference)	1.00 (Reference)	1.00 (Reference)	1.00 (Reference)
	Q2	131/10,663	2.51	1.16 (0.90–1.49)	1.08 (0.84–1.39)	1.02 (0.79–1.32)	0.98 (0.75–1.26)
	Q3	174/10,663	3.38	1.56 (1.23–1.97)***	1.43 (1.13–1.81)**	1.32 (1.04–1.69)*	1.18 (0.91–1.52)
	Q4	254/10,663	5.01	2.30 (1.85–2.87)***	2.10 (1.69–2.63)***	1.93 (1.53–2.43)***	1.44 (1.10–1.88)**
CHD	Per 1 unit	732/42,651		1.28 (1.15–1.44)***	1.23 (1.09–1.38)***	1.13 (0.99–1.28)	0.99 (0.85–1.15)
	Q1	149/10,662	2.80	1.00 (Reference)	1.00 (Reference)	1.00 (Reference)	1.00 (Reference)
	Q2	168/10,663	3.22	1.15 (0.93–1.44)	1.05 (0.84–1.31)	1.00 (0.80–1.25)	0.93 (0.74–1.17)
	Q3	191/10,663	3.71	1.33 (1.07–1.65)**	1.18 (0.95–1.46)	1.08 (0.87–1.35)	0.92 (0.73–1.16)
	Q4	224/10,663	4.41	1.58 (1.29–1.95)***	1.41 (1.14–1.73)***	1.23 (0.99–1.53)	0.88 (0.70–1.13)

Exact values of quartiles of TyG index are as follows: (Q1) ≤ 8.246, (Q2) ≤ 8.609 and > 8.246, (Q3) ≤ 9.020 and > 8.609, and (Q4) > 9.020

*: *P* < 0.05; **: *P* < 0.01; ***: *P* < 0.001

Model 1: unadjusted model

Model 2: adjusted for age and sex

Model 3: adjusted for age, sex, body mass index, education level, physical activity, current smoking and current drinking

Model 4: adjusted for age, sex, body mass index, education level, physical activity, current smoking, current drinking, high-density lipoprotein cholesterol, uric acid, antihypertensive medication and antidiabetic medication

CHD, coronary heart disease; CI, confidence interval; CVD, cardiovascular disease; Q, quartiles; TyG, triglyceride-glucose

*P* for trend was calculated by modelling TyG index as a continuous variable into Cox proportional model

**Fig. 3:**
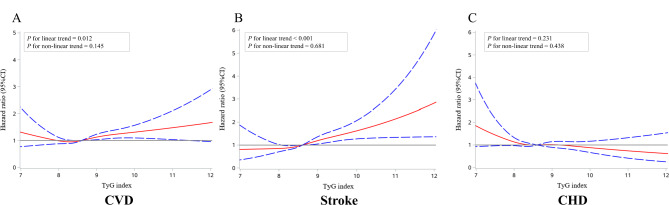
Multivariable-adjusted restricted cubic splines of the association between TyG index and cardiovascular disease. CHD, coronary heart disease; CI, confidence interval; CVD, cardiovascular disease; TyG, triglyceride-glucose. Hazard ratios were adjusted for age, sex, body mass index, education level, physical activity, current smoking, current drinking, systolic blood pressure, low-density lipoprotein cholesterol, high-density lipoprotein cholesterol, uric acid, and antihypertensive medication

Multivariable-adjusted restricted cubic splines were presented in Fig. 3. Linear associations of TyG index with CVD (*P* for linearity = 0.012) and with stroke (*P* for linearity < 0.001) were observed. There was no evidence of non-linear relationships between TyG index and CVD or stroke. CHD had neither linear nor non-linear relationships with TyG index.

### Subgroup analysis

In order to determine the association between TyG index with incident CVD, stroke, and CHD more clearly, subgroup analyses were conducted (Table 3). We found that in the younger age group (< 50 years) TyG index was associated with a higher risk of CVD than in the older group (≥ 50 years) (*P* for interaction = 0.002). This similar trend in the younger group was generally observed for stroke and CHD. Compared to the hypertensive, the non-hypertensive subgroup had a slightly higher risk of CVD, stroke and CHD (*P* for interactions = < 0.001, 0.008, and 0.004, respectively) per 1 increment increase in TyG index, although each group may not have individually reached statistical significance for some of the events. With regards to diabetes, TyG was associated with a higher risk of CVD and stroke (*P* for interactions =  < 0.001 and 0.001, respectively) in the diabetic group compared to the non-diabetic group. Although an interaction was observed for CHD events (*P* for interaction = 0.007), the direction of the relationship is unclear. TyG index showed a trend of higher risk of CVD and CHD (*P* for interactions = 0.007 and 0.008, respectively) in participants without dyslipidemia, compared to those with dyslipidemia. A similar trend for stroke was seen, although not significant (*P* for interaction = 0.277). No obvious differences in the relationship of TyG with the different CVD outcomes in sex or BMI subgroups was observed (*P* for interactions > 0.05).

**Table 3:** Association between TyG index with the risk of cardiovascular disease according to subgroups

		CVD	*P* for interaction	Stroke	*P* for interaction	CHD	*P* for interaction
Subgroups	N	HR (95% CI)	*P*	HR (95% CI)	*P*	HR (95% CI)	*P*
Sex			0.555		0.919		0.967
Male	17,208	1.22 (1.06–1.41)	0.006	1.40 (1.16–1.70)	< 0.001	0.93 (0.75–1.15)	0.486
Female	25,447	1.08 (0.92–1.28)	0.342	1.35 (1.06–1.71)	0.015	0.85 (0.68–1.07)	0.157
Age			0.002		0.066		0.008
< 50	9,924	1.64 (1.12–2.42)	0.012	1.89 (1.09–3.26)	0.023	1.55 (0.83–2.86)	0.166
≥ 50	32,731	1.12 (1.00-1.25)	0.055	1.34 (1.15–1.56)	< 0.001	0.84 (0.71–0.99)	0.030
BMI			0.399		0.263		0.954
< 18.5	2,586	0.62 (0.20–1.94)	0.409	1.05 (0.25–4.41)	0.947	0.22 (0.04–1.33)	0.100
18.5–23.9	19,206	1.25 (1.04–1.50)	0.002	1.66 (1.29–2.12)	< 0.001	0.88 (0.67–1.16)	0.370
24.0-27.9	15,761	1.09 (0.93–1.27)	0.290	1.17 (0.93–1.46)	0.179	0.91 (0.73–1.14)	0.395
≥ 28	5,102	1.19 (0.92–1.54)	0.181	1.46 (1.04–2.04)	0.028	0.81 (0.56–1.17)	0.265
Hypertension			< 0.001		0.008		0.004
Yes	21,346	1.10 (0.97–1.24)	0.136	1.32 (1.11–1.57)	0.002	0.83 (0.69–0.99)	0.039
No	21,309	1.20 (0.97–1.49)	0.097	1.44 (1.06–1.95)	0.019	0.95 (0.69–1.29)	0.733
Diabetes			< 0.001		0.001		0.007
Yes	4,358	1.23 (1.02–1.48)	0.030	1.53 (1.20–1.95)	< 0.001	0.84 (0.63–1.12)	0.224
No	38,297	1.04 (0.91–1.19)	0.596	1.17 (0.96–1.42)	0.124	0.84 (0.69–1.01)	0.070
Dyslipidemia			0.007		0.277		0.008
Yes	14,773	1.02 (0.87–1.18)	0.836	1.28 (1.04–1.58)	0.020	0.71 (0.57–0.89)	0.002
No	27,882	1.17 (0.97–1.41)	0.112	1.43 (1.09–1.88)	0.010	0.91 (0.70–1.18)	0.473

h present risks in each subgroup per 1 unit higher TyG index

BMI, body mass index; CHD, coronary heart disease; CI, confidence interval; CVD, cardiovascular disease; HR, hazard ratio; TyG, triglyceride-glucose

HRs were adjusted for age, sex, body mass index, education level, physical activity, current smoking, current drinking, high-density lipoprotein cholesterol, uric acid, antihypertensive medication and antidiabetic medication

Subgroup analysis of ASCVD was conducted to determine whether different types of stroke had different patterns of association with TyG index (Table S3). A similar trend was observed in ASCVD and ischaemic stroke compared to the association with CVD and stroke: higher TyG index was correlated with higher risk of ASCVD [1.16 (1.04–1.29)] and ischaemic stroke [1.47 (1.24–1.76)]. No relationship between TyG index and haemorrhagic stroke was observed.

## Discussion

In this large, community-based natural cohort study, it was found that an increased TyG index was associated with a higher risk of developing CVD. The associations with CVD and stroke remained statistically significant after adjustments for potential confounders, such as established cardiovascular risk factors. With each unit increment in TyG index, participants had 16% and 39% increased risk of developing CVD and stroke, respectively, independent of potential confounders. The relationships were more pronounced in participants under 50 years old, those without hypertension and those with diabetes, suggesting that a high TyG index could be a strong predictor of CVD events in these groups.

### TyG and CVD

Several previous studies also have demonstrated a positive association between TyG index and CVD events. A 10-year follow-up cohort study, the VMCUN cohort included a total number of 5,014 outpatients aged 18 to 90 years, found that participants within the highest quintile of TyG index had a 32% increased risk of developing CVD compared to those in the lowest quintile, after adjustment for potential confounders [14]. In a retrospective observational cohort study of 5,593,134 participants aged ≥ 40 years from the Korea National Health Information Database (NHID) with an 8.2-year follow-up, Hong et al. revealed that patients in the highest TyG index quartile were related to a higher risk of CVD [1.28 (1.26–1.30)], stroke [1.26 (1.23–1.29)], and CHD [1.31 (1.28–1.35)] [22]. Furthermore, results from the Kailuan study that consisted of over 76% male miner workers in China included 49,579 participants during a median follow-up time of 9 years showed that the highest tertile of TyG index was associated with higher CVD incidence compared with the lowest tertile [1.25 (1.11–1.42)] [16]. However, these studies either included outpatients or specific populations or were retrospective cohort studies. Our study was a prospective study based on a general population in China with a large sample size, both men and women and a broad age range (20 to 74 years), which is more generalizable than other studies. Consistent with previous studies, our study confirmed that higher TyG index was significantly related with increased risk of total CVD and stroke. However, different from some other studies [16, 23], TyG index was not associated with CHD in our population after full covariate adjustment. In Li et al.’s study [16], although they had adjusted for many of the same covariates as our study in their final models, they did not include important potential confounders, such as use of antihypertensive or antidiabetic medication. Tian et al. [23] had similar covariates, but instead had included use of antidiabetic, lipid-lowering, and antihypertensive medication. While inclusion of medication may not be the reason, possible explanations for differences from our study findings were that the studies by Li et al. and Tian et al. had more CHD cases and a longer follow-up time (median 11.0 years) than our study and that most of their participants were male (79.8%). In subgroup analysis of subtypes of stroke, we found higher TyG index was more related with ischaemic stroke. The mechanisms of developing stroke (ischaemic and heamorrhagic stroke) and CHD are different. CHD is due to atherosclerosis and thrombosis in the arteries [24], while ischaemic stroke is more related with embolism, which can from atherosclerotic plaque in arteries or aortic arch or heart [25]. Haemorrhagic stroke is mostly caused by deep perforating vasculopathy, which is associated with high blood pressure [26]. As TyG index encompasses triglyceride and glucose values and is a surrogate marker of IR, it would make sense that it is more strongly related to CVD events, such as ischaemic stroke. IR is associated with endothelium dysfunction [27, 28] and promotes the vulnerable atherosclerotic plaque [29], which might contribute to ischaemic stroke. Moreover, TyG index is proved to be related with arterial stiffness [30] and glucose metabolism [31], the major risk factor of CHD. However in our final model, there was no relationship between TyG index and CHD. More studies should be conducted to clarify the association between TyG index and CHD.

### Relationship of TyG with CVD in different subgroups

We found that among participants under 50 years old, each unit increment in TyG index was associated with 64% and 89% in risk of future CVD and stroke, which was more pronounced than in those over 50 years old. A similar trend was observed with stroke and CHD. However, these results may be due to the small sample size of the group under 50 years old. Up to now, most studies assessing the relationship between TyG with CVD were conducted in middle-aged or elderly populations [13, 22]. Thus, more studies on TyG index and CVD events in younger individuals should be conducted. Hypertension, diabetes, and dyslipidemia have been found to accelerate the progression of atherosclerosis and CVD [32]. IR, a pathway of developing hypertension, diabetes, and dyslipidemia, appears before diseases are diagnosed [32, 33]. In our subgroup analysis, we found that individuals without hypertension had a slightly higher risk of developing stroke compared to those with hypertension. Similar results were found by Hong et al.: in the highest TyG index quartile, hypertensive individuals have a lower risk of CVD [1.12 (1.10–1.14)] and stroke [1.10 (1.08–1.13)] than non-hypertensive individuals [CVD: 1.21 (1.19–1.23); stroke: 1.20 (1.18–1.13)] [22]. In our present analysis, each unit increment in TyG index was associated with a higher risk of CVD and stroke in participants with diabetes, compared to those without diabetes. In contrast, TyG index was associated with a higher risk of stroke in those without dyslipidemia, compared to those with dyslipidemia, although this might be due to overadjustment in the final model, such as HDL-C and antidiabetic medication. The results from the UK Biobank including 403,335 individuals elucidated that the positive association between baseline TyG index and CVD was largely mediated by dyslipidemia, type 2 diabetes, and hypertension [34]. Hypertension, diabetes, and dyslipidemia are currently the most prevalent co-morbidities around the world [35]. Participants in SSACB, from our study, had a high rate of hypertension (50%) and antihypertensive medication use (27.6%). Although we adjusted for antihypertensive and diabetic medication use, TyG index might also be affected by the use of other medications, such as for dyslipidemia, which we had no data on.

While we found no statistically meaningful difference between men and women in our study (no evidence of interaction), a meta-analysis demonstrated that women in the highest quartile of TyG index [1.65 (1.13–2.42)] had a higher risk of CVD than men [1.44 (1.14–1.83)] [15] compared to those in the lowest quartile. In our study, we did, however, observe slightly higher associations of TyG with CVD in men compared to women. Although differences in TyG index between men and women are still uncertain, men do tend to have a higher prevalence of lifestyle factors known to lead to metabolic diseases, such as smoking and drinking [36], which can affect the HR estimates observed. Possible reasons for discrepancies between our study and others, is that we adjusted for covariates that do not cover the entire range of smoking and drinking amounts (only for drinking status and smoking status). Moreover, our study is based on a general population encompassing a broad age-range and with a different ethnicity (Chinese population), which leads to a population that is distinct from those of previous research.

### Potential mechanisms

Potential mechanisms that contribute to the predictive role of TyG index with future CVD remain unclear. It is clear that TyG index, consisting of TG and FPG, lipid-related and glucose-related CVD risk factors, reflect IR in the human body. Firstly, IR results in glucose metabolism imbalance, which causes chronic inflammation, oxidative stress, and lipid disturbances. These may initiate the progression of atherosclerosis [37]. Secondly, it has been reported that IR can cause endothelial dysfunction [27, 28, 38], by inducing higher levels of glycosylated products and free radicals, leading to nitric oxide (NO) inactivation, and resulting in endothelium-dependent vasodilation [39]. Thirdly, studies have illustrated that IR can contribute to platelet hyperactivity, the increase in adhesion-induced and thromboxane A2 (TxA2)-dependent tissue factor expression in platelets, and aggregation [40–42], which may explain artery stenosis or occlusion. Fourthly, IR, usually accompanied by hyperglycemia, has been found to induce excessive glycosylation which promotes smooth muscle cell proliferation and collagen crosslinking and deposition. This results in increased ventricular stiffness, cardiac fibrosis, and, ultimately, CVD events [43].

### Strengths and limitations

The strengths of our study included a large sample size of a general population with a broad age-range (which includes young adults) and a longitudinal study design. Furthermore, our dataset had complete linkage to medical record systems which were used for the ascertainment of events (CVD, stroke, and CHD), as well as for accurate double-checks of the self-reported medical history. However, our study had several limitations. Firstly, because the follow-up time of our study was relatively short (4.7 years), the total CVD cases (1,422 cases) might not have been sufficient for the detection of true associations in some of the stratified analyses. For this reason, long-term follow-up studies should be conducted to determine the relationships between TyG index with CVD events. Secondly, TyG index was only obtained once at baseline, time-varying changes during the follow-up time could not be considered in our analysis, which may lead to potential bias. Thirdly, due to the observational design of our study, although we adjusted for several major confounders, residual confounding effects could not be completely excluded, for example, a higher TyG index is associated with a higher prevalence of many lifestyle factors that are well-known strong risk factors for CVD.

## Conclusion

An elevated TyG index was associated with a higher risk of incident CVD and stroke, especially in younger and diabetic populations. Moreover, the association of TyG with CVD is generally more pronounced in non-hypertensive and non-dyslipidemic groups. Thus, TyG index, as a surrogate marker of IR, may help in the early stage of identifying people at high risk of CVD and may be useful in its primary prevention.

### Electronic supplementary material

Below is the link to the electronic supplementary material.

Supplementary Material 1

## Data Availability

The datasets generated and analyzed in the current study are not publicly available due to confidentiality but are available from the corresponding author at reasonable request.

## List of abbreviations

ASCVD: Atherosclerotic cardiovascular disease
BMI: Body Mass Index
CCDRR: Cardiovascular and Cerebrovascular Disease Registration and Reporting System
CDSS: Cause-of-Death Surveillance System
CHD: Coronary heart disease
CI: Confidence interval
CVD: Cardiovascular disease
DBP: Diastolic blood pressure
EMR: Electronic Medical Record System
FPG: Fasting plasma glucose
HbA1c: Glycated hemoglobin
HDL-C: High-density lipoprotein cholesterol
HOMA-IR: Homeostasis model assessment estimated insulin resistance
HR: Hazard ratio
ICD-10: International Statistical Classification of Diseases and Related Health Problems, Tenth Revision
IPAQ: International Physical Activity Questionnaire
IR: Insulin resistance
LDL-C: Low-density lipoprotein cholesterol
MET: Metabolic equivalent
MS: Metabolic syndrome
NHID: National Health Information Database
NO: Nitric oxide
RCS: Restricted cubic spline
SBP: Systolic blood pressure
SD: Standard deviation
SSACB: Shanghai Suburban Adult Cohort and Biobank
TC: Total cholesterol
TG: Triglyceride
TxA2: Thromboxane A2
TyG: Triglyceride-glucose
VMCUN: Vascular Metabolic CUN
