# Supplementary material for: Association of triglyceride-glucose index with cardiovascular disease among a general population: a prospective cohort study
Source: Diabetol Metab Syndr. 2023 Oct 16;15:204. doi: 10.1186/s13098-023-01181-z (PMC10580532; doi:10.1186/s13098-023-01181-z)
Supplement: Supplementary file 1 — Supplementary Material 1 [file 13098_2023_1181_MOESM1_ESM.docx]

**Table S1.** Baseline characteristics of the study population according to CVD, stroke, CHD cases and healthy participants

| **Characteristics^a^** | **Total** | **CVD cases** | **Stroke cases** | **CHD cases** | **Healthy participants** |
| --- | --- | --- | --- | --- | --- |
| N | 42,651 | 1,422 | 674 | 732 | 41,229 |
| Female | 25,447 (59.7) | 678 (47.7) | 300 (44.5) | 384 (52.5) | 24,769 (60.1) |
| Age, years | 55.7 ± 11.1 | 62.7 ± 7.4 | 62.8 ± 7.4 | 62.7 ± 7.3 | 55.5 ± 11.1 |
| Education level |  |  |  |  |  |
| Primary school and below | 5,181 (12.2) | 261 (18.4) | 114 (16.9) | 147 (20.1) | 4,919 (11.9) |
| Middle school | 12,422 (29.1) | 597 (42.0) | 278 (41.3) | 309 (42.2) | 11,825 (28.7) |
| High school or above | 25,049 (58.7) | 564 (39.7) | 282 (41.8) | 276 (37.7) | 24,485 (59.4) |
| Current smoking | 10,395 (24.4) | 472 (33.2) | 247 (36.7) | 206 (28.1) | 9,923 (24.1) |
| Current drinking | 5,758 (13.5) | 267 (18.8) | 136 (20.2) | 526 (71.9) | 5,491 (13.3) |
| Physical activity level |  |  |  |  |  |
| Low | 4,173 (9.8) | 142 (10.0) | 74 (11.0) | 64 (8.7) | 4,031 (9.8) |
| Moderate | 13,803 (32.4) | 420 (29.5) | 211 (31.3) | 201 (27.5) | 13,383 (32.5) |
| High | 24,675 (57.9) | 860 (60.5) | 389 (57.7) | 467 (63.8) | 23,815 (57.8) |
| Body mass index, kg/m^2^ | 24.2 ± 3.3 | 25.1 ± 3.4 | 25.1 ± 3.4 | 25.2 ± 3.5 | 24.2 ± 3.3 |
| Systolic blood pressure, mmHg | 132.8 ± 19.3 | 140.3 ± 20.6 | 142.8 ± 21.4 | 138.0 ± 19.2 | 132.5 ± 19.2 |
| Diastolic blood pressure, mmHg | 79.6 ± 10.6 | 82.0 ± 10.8 | 82.8 ± 11.3 | 81.3 ± 10.3 | 79.6 ± 10.6 |
| Uric acid, mg/dL | 303.8 ± 80.7 | 323.6 ± 86.0 | 324.3 ± 85.3 | 321.7 ± 87.0 | 303.1 ± 80.4 |
| Fasting plasma glucose, mmol/L | 4.9 (4.4, 5.5) | 5.1 (4.4, 6.0) | 5.2 (4.5, 6.2) | 5.0 (4.3, 5.6) | 4.9 (4.4, 5.5) |
| Triglycerides, mmol/L | 1.4 (1.0, 2.0) | 1.5 (1.1, 2.2) | 1.6 (1.1, 2.2) | 1.5 (1.1, 2.1) | 1.4 (1.0, 2.0) |
| Total cholesterol, mmol/L | 4.9 ± 0.9 | 5.0 ± 1.0 | 5.1 ± 1.1 | 5.0 ± 1.0 | 4.9 ± 0.9 |
| High-density lipoprotein cholesterol, mmol/L | 1.4 ± 0.4 | 1.3 ± 0.4 | 1.3 ± 0.4 | 1.4 ± 0.4 | 1.4 ± 0.4 |
| Low-density lipoprotein cholesterol, mmol/L | 2.8 ± 0.8 | 2.9 ± 0.9 | 2.9 ± 0.9 | 2.8 ± 0.9 | 2.8 ± 0.8 |
| HbA1c, % | 5.70 (5.4, 6.0) | 5.8 (5.5, 6.4) | 5.9 (5.6, 6.5) | 5.8 (5.5, 6.2) | 5.7 (5.4, 6.0) |
| Triglycerides-glucose index | 8.6 (8.3, 9.0) | 8.8 (8.4, 9.2) | 8.8 (8.4, 9.3) | 8.7 (8.3, 9.1) | 8.6 (8.2, 9.0) |
| Hypertension | 21,342 (50.0) | 1.091 (76.7) | 488 (72.4) | 525 (71.7) | 20,324 (49.3) |
| Antihypertensive medication | 11,786 (27.6) | 709 (49.9) | 341 (50.6) | 368 (50.3) | 11,077 (26.9) |
| Diabetes | 4,357 (10.2) | 331 (23.3) | 180 (26.7) | 150 (20.5) | 4,026 (9.8) |
| Antidiabetic medication | 4,538 (10.6) | 348 (24.5) | 184 (27.3) | 158 (21.6) | 4,190 (10.2) |
| Dyslipidemia | 14,770 (34.6) | 650 (45.7) | 313 (46.4) | 322 (44.0) | 14,120 (34.2) |

CHD, coronary heart disease; CVD, cardiovascular disease; N, number; Q, quartiles; TyG, triglyceride-glucose; HbA1c, glycated hemoglobin A1c.

^a^ Continuous variables were described as mean ± standard deviation (SD); Categorical variables were described as frequency (percentage).

**Table S2.** Sensitivity analysis: Association between TyG index and incidence of cardiovascular diseases

| **Event** | **TyG** | **No. of cases/population^a^** | **Incidence rate per 1000 person-years** | **Hazard ratio (95% CI)** | | | |
| --- | --- | --- | --- | --- | --- | --- | --- |
|  |  |  |  | **Model 1** | **Model 2** | **Model 3** | **Model 4** |
| CVD | Per 1 unit | 1,160/42,389 |  | 1.56 (1.43-1.70)*** | 1.52 (1.39-1.66)*** | 1.45 (1.31-1.59)*** | 1.16 (1.03-1.31)* |
|  | Q1 | 222/10,598 | 4.20 | 1.00 (Reference) | 1.00 (Reference) | 1.00 (Reference) | 1.00 (Reference) |
|  | Q2 | 228/10,597 | 4.41 | 1.05 (0.88-1.27) | 0.97 (0.81-1.17) | 0.92 (0.76-1.11) | 0.87 (0.71-1.05) |
|  | Q3 | 300/10,597 | 5.88 | 1.41 (1.18-1.68)*** | 1.27 (1.07-1.51)** | 1.18 (0.98-1.41) | 1.02 (0.84-1.23) |
|  | Q4 | 410/10,597 | 8.16 | 1.96 (1.66-2.31)*** | 1.76 (1.50-2.08)*** | 1.60 (1.34-1.90)*** | 1.15 (0.94-1.40) |
|  |  |  |  |  |  |  |  |
| Stroke | Per 1 unit | 525/42,389 |  | 1.75 (1.54-1.98)*** | 1.72 (1.51-1.96)*** | 1.67 (1.45-1.91)*** | 1.33 (1.13-1.58)*** |
|  | Q1 | 93/10,598 | 1.75 | 1.00 (Reference) | 1.00 (Reference) | 1.00 (Reference) | 1.00 (Reference) |
|  | Q2 | 97/10,597 | 1.87 | 1.07 (0.80-1.42) | 0.99 (0.75-1.32) | 0.94 (0.71-1.26) | 0.89 (0.66-1.19) |
|  | Q3 | 135/10,597 | 2.63 | 1.50 (1.15-1.96)** | 1.38 (1.06-1.80)* | 1.27 (0.97-1.67) | 1.10 (0.82-1.46) |
|  | Q4 | 200/10,597 | 3.95 | 2.26 (1.77-2.89)*** | 2.06 (1.61-2.63)*** | 1.90 (1.46-2.46)*** | 1.32 (0.98-1.79) |
|  |  |  |  |  |  |  |  |
| CHD | Per 1 unit | 614/42,389 |  | 1.32 (1.17-1.50)*** | 1.27 (1.11-1.44)** | 1.17 (1.02-1.34)* | 0.95 (0.80-1.12) |
|  | Q1 | 128/10,598 | 2.41 | 1.00 (Reference) | 1.00 (Reference) | 1.00 (Reference) | 1.00 (Reference) |
|  | Q2 | 131/10,597 | 2.52 | 1.05 (0.82-1.34) | 0.95 (0.75-1.22) | 0.90 (0.70-1.16) | 0.85 (0.66-1.10) |
|  | Q3 | 162/10,597 | 3.16 | 1.32 (1.05-1.66)* | 1.17 (0.93-1.47) | 1.08 (0.85-1.40) | 0.95 (0.74-1.22) |
|  | Q4 | 193/10,597 | 3.81 | 1.60 (1.28-2.00)*** | 1.41 (1.13-1.77)** | 1.24 (0.98-1.57) | 0.94 (0.71-1.24) |

Exact values of quartiles of TyG index are as follows: (Q1)≤8.246, (Q2)≤8.609 and >8.246, (Q3)≤9.020 and >8.609, and (Q4) >9.020.

^a^: CVD events occurred within the first year of follow-up were excluded.

*: *P* < 0.05; **: *P* < 0.01; ***: *P* < 0.001.

Model 1: an unadjusted model.

Model 2: adjusted for age and sex.

Model 3: adjusted for age, sex, body mass index, education level, physical activity, current smoking and current drinking.

Model 4: adjusted for age, sex, body mass index, education level, physical activity, current smoking, current drinking, high-density lipoprotein cholesterol, uric acid, antihypertensive medication and antidiabetic medication.

CHD, coronary heart disease; CI, confidence interval; CVD, cardiovascular disease; Q, quartiles; TyG, triglyceride-glucose.

*P* for trend was calculated by modelling TyG index as a continuous variable into Cox proportional model.

**Table S3.** Subgroup analysis: Association between TyG index and incidence of atherosclerotic cardiovascular diseases

| **Event** | **TyG** | **No. of cases/population** | **Incidence rate per 1000 person-years** | **Hazard ratio (95% CI)** | | | |
| --- | --- | --- | --- | --- | --- | --- | --- |
|  |  |  |  | **Model 1** | **Model 2** | **Model 3** | **Model 4** |
| ASCVD | Per 1 unit | 1,355/42,651 |  | 1.56 (1.44-1.69)*** | 1.52 (1.40-1.65)*** | 1.45 (1.33-1.59)*** | 1.16 (1.04-1.29)** |
|  | Q1 | 249/10,662 | 4.70 | 1.00 (Reference) | 1.00 (Reference) | 1.00 (Reference) | 1.00 (Reference) |
|  | Q2 | 280/10,663 | 5.41 | 1.15 (0.97-1.36) | 1.06 (0.89-1.26) | 1.01 (0.85-1.20) | 0.95 (0.80-1.13) |
|  | Q3 | 349/10,663 | 6.83 | 1.45 (1.24-1.71)*** | 1.32 (1.12-1.55)** | 1.23 (1.04-1.46)* | 1.05 (0.88-1.26) |
|  | Q4 | 477/10,663 | 9.49 | 2.02 (1.73-2.36)*** | 1.83 (1.57-2.13)*** | 1.66 (1.42-1.96)*** | 1.18 (0.98-1.42) |
|  |  |  |  |  |  |  |  |
| Ischaemic stroke | Per 1 unit | 471/42,651 |  | 1.88 (1.65-2.14)*** | 1.87 (1.64-2.14)*** | 1.85 (1.60-2.12)*** | 1.47 (1.24-1.76)*** |
|  | Q1 | 71/10,662 | 1.33 | 1.00 (Reference) | 1.00 (Reference) | 1.00 (Reference) | 1.00 (Reference) |
|  | Q2 | 90/10,663 | 1.72 | 1.29 (0.94-1.76) | 1.21 (0.89-1.65) | 1.16 (0.84-1.59) | 1.09 (0.79-1.51) |
|  | Q3 | 121/10,663 | 2.34 | 1.75 (1.31-2.35)*** | 1.62 (1.21-2.18)** | 1.56 (1.16-2.11)** | 1.35 (0.99-1.85) |
|  | Q4 | 189/10,663 | 3.71 | 2.77 (2.11-3.63)*** | 2.56 (1.95-3.36)*** | 2.45 (1.84-3.26)*** | 1.69 (1.22-2.35)** |
|  |  |  |  |  |  |  |  |
| Haemorrhagic stroke | Per 1 unit | 67/42,651 |  | 1.28 (0.88-1.87) | 1.23 (0.84-1.82) | 1.01 (0.67-1.54) | 0.91 (0.57-1.45) |
|  | Q1 | 13/10,662 | 0.24 | 1.00 (Reference) | 1.00 (Reference) | 1.00 (Reference) | 1.00 (Reference) |
|  | Q2 | 16/10,663 | 0.30 | 1.25 (0.60-2.60) | 1.16 (0.56-2.42) | 1.03 (0.50-2.16) | 1.05 (0.50-2.21) |
|  | Q3 | 19/10,663 | 0.37 | 1.50 (0.74-3.04) | 1.37 (0.68-2.78) | 1.04 (0.51-2.15) | 1.07 (0.51-2.28) |
|  | Q4 | 19/10,663 | 0.37 | 1.51 (0.75-3.06) | 1.37 (0.68-2.78) | 0.99 (0.48-2.04) | 1.07 (0.48-2.40) |

Exact values of quartiles of TyG index are as follows: (Q1)≤8.246, (Q2)≤8.609 and >8.246, (Q3)≤9.020 and >8.609, and (Q4) >9.020.

*: *P* < 0.05; **: *P* < 0.01; ***: *P* < 0.001.

Model 1: an unadjusted model.

Model 2: adjusted for age and sex.

Model 3: adjusted for age, sex, body mass index, education level, physical activity, current smoking and current drinking.

Model 4: adjusted for age, sex, body mass index, education level, physical activity, current smoking, current drinking, high-density lipoprotein cholesterol, uric acid, antihypertensive medication and antidiabetic medication.

ASCVD, atherosclerotic cardiovascular diseases; CHD, coronary heart disease; CI, confidence interval; CVD, cardiovascular disease; Q, quartiles; TyG, triglyceride-glucose.

*P* for trend was calculated by modelling TyG index as a continuous variable into Cox proportional model.
